# Revealing novel insights into the improvement of greenhouse tea quality through exogenous substance interventions using targeted and untargeted metabolomics and microbial community analyses

**DOI:** 10.1016/j.fochx.2025.102410

**Published:** 2025-03-24

**Authors:** Haozhen Li, Shuyao Wang, Xiaohua Zhang, Kangkang Song, Long Yang

**Affiliations:** a College of Plant Protection, Agricultural Big-Data Research Center and Key Laboratory of Agricultural Film Application of Ministry of Agriculture and Rural Affairs, Shandong Agricultural University, Tai'an 271018, China; b Bioresource Engineering, McGill University, Sainte-Anne-de-Bellevue, QC H9X 3V9, Canada

**Keywords:** Quality, Greenhouse tea, Metabolomics, Microorganisms

## Abstract

Tea quality in greenhouse was certain gap with open air. Metabolites and foliar microorganisms were investigated under seaweed fertiliser (CF) and gibberellin (CH) treatments using sensory evaluation, HPLC, untargeted metabolomics, 16S rDNA, and Internal Transcribed Spacer. CF tea was mellow, less astringent, and of better quality compared to CH. Catechin, −(−)Epicatechin, and Epigallocatechin were notably lower in CF. Differentially accumulated metabolites (DAMs) were notably enriched in Flavonoid and Phenylpropanoid biosynthesis, both involved in Catechin synthesis. DAMs in these pathways appeared down-regulated in CF. The CF improved quality by down-regulating metabolites in Phenylpropanoid biosynthesis in conjunction with microbial community metabolism enriched in amino acid and secondary metabolite biosynthesis. Metabolite- microbial correlation analysis indicated that the highest correlation with phenylpropane pathway metabolites was in bacteria *Variovorax* and *Pseudomonas*, and in fungi *Filobasidium*. The study provides theoretical basis for regulating flavour quality of greenhouse tea.

## Highlights

• CCF exhibited umami, mellow and fragrant and sweet flavour, while CH showed slightly astringent, mellow and thick
• Catechin, −(−)Epicatechin, and Epigallocatechin flavour metabolites were notably lower in CF
• Phenylpropanoid biosynthesis was identified as a key pathway driving the flavour differences
• Strong correlations were observed between signature microorganisms and flavour substances
• A greenhouse tea quality improvement measure was proposed

## Graphical abstract

**Figure:**
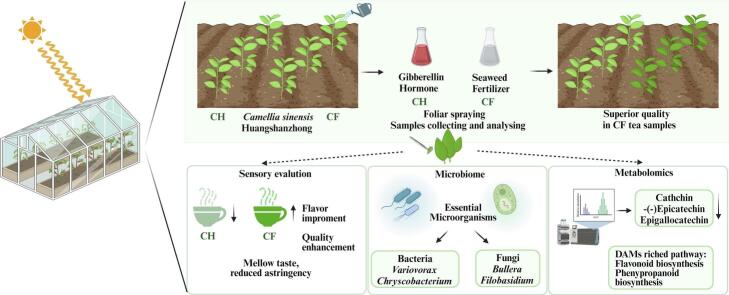

## Chemical compounds studied in the article

Catechin (PubChem CID 9064)

(−)-Epicatechin (PubChem CID 72276)

Epigallocatechin (PubChem CID 72277)

Gallocatechin (PubChem CID 65084)

(−)-Catechin gallate (PubChem CID 6419835)

(−)-Epicatechin gallate (PubChem CID 107905)

(−)-Epigallocatechin gallate (PubChem CID 65064)

Gallocatechin gallate (PubChem CID 199472)

Caffeine (PubChem CID 2519)

L-Theanine (PubChem CID 439378).

## 1 Introduction

Tea plants are normally cultivated in tropical and subtropical regions (Wang et al., 2020) and are susceptible to low temperatures in mid- to high-latitudes regions and at high altitudes (Tong et al., 2024). Winter freezes or spring frosts can cause massive deaths of tea plants (Di et al., 2024). Some regions in northern China are still subject to spring cold affecting tea plants even into March (Hao et al., 2018). In order to safely survive the winter and prevent spring cold, plastic greenhouse facilities are used to artificially control environmental factors such as temperature and humidity, promoting tea plants growth or breaking dormancy to allow for earlier harvest. Inside the greenhouse, temperatures are warmer, and the humidity is higher (Li et al., 2016), leading to faster leaf development, with elongated buds and thinner leaves. However, the accumulation of key tea compounds, particularly quality-related metabolites, may be limited. After brewing, the fresh flavour remains fine, but the taste is bland, and the aroma is common (Ma et al., 2024). Appearance of both the dry tea and the leaf bottom is clearly distinct from the of tea grown in the open air.

In traditional tea garden management, gibberellin (GA) significantly promotes the spring sprouting of overwintering tea plants by activating the phenylpropanoid biosynthesis pathway (Di et al., 2019), accelerating internode elongation and leaf expansion (Yue et al., 2018). However, the increase in yield may lead to an imbalance in quality, prompting researchers to explore more comprehensive tea garden management strategies. Meanwhile, the accumulation of various seaweeds along China's coastline is often treated as waste (Civelek Yoruklu et al., 2022). These seaweeds are rich in organic compounds, serving as a natural biological resource for organic fertilizers (Gibilisco et al., 2022). Foliar application of seaweed extracts has been shown to enhance crop quality and growth (Shang et al., 2024), yet their potential in tea cultivation remains underexplored.

High quality tea is highly regarded for its smooth taste, low bitterness and astringency, and rich umami flavour. However, the bitter and astringent notes of tea remain unappealing to many consumers. For instance, flavonoid glycosides and catechin compounds are the primary contributors to bitterness and astringency (Ye et al., 2022). Flavour, as a key indicator of tea quality (Zhang et al., 2020), is largely determined by the composition and content of non-volatile compounds (Wang et al., 2022). These compounds not only shape the unique flavour profile of tea but also influence its leaf morphology, colour, aroma, and functional properties (Wang et al., 2021). In addition, microorganisms play a crucial role in the formation of tea flavour (Han et al., 2024). The involvement of microbes can impart characteristic flavours to tea (Tian et al., 2024). For example, the collaboration between bacteria and fungi promotes dynamic changes in flavonoid compounds and amino acids (Zhang et al., 2024), and the enzymes they secrete can catalyze the metabolic transformation of important secondary metabolites. In different types of tea, microbial activities exhibit diverse characteristics. In the production of Fu brick tea, core functional microorganisms significantly reduce the content of catechins and flavonoid compounds, thereby lowering bitterness and astringency while enhancing mellowness (Li et al., 2021a). In Wuyi rock tea, the bacterium *Chryseobacterium* influences the production of catechin compounds, shaping the distinctive rock flavour (Wu et al., 2024). In Liubao tea, *Sphingomonas* improves flavour by modulating the biosynthesis of flavonoids and flavonols (Liu et al., 2024). Furthermore, in Shui Xian and Pu’er teas, microorganisms regulate metabolic products to create unique flavour profiles (He et al., 2023; Yuan et al., 2024). Therefore, it is essential to study the impact of microorganisms on the quality of greenhouse tea.

To mitigate quality deficiencies in greenhouse tea due to faster growth. In this study, sensory evaluation, HPLC, metabolites with 16S and Internal Transcribed Spacer (ITS) techniques were used to study the gap between microbiome and metabolites due to seaweed fertiliser and gibberellin treatments and explored the best model suitable for quality improvement of greenhouse tea plants. Changes, effects, and interactions patterns of tea leaves microbes and metabolites were analysed. The key metabolites and core microorganisms responsible for improving tea quality were identified, and their functions were fully explored, with the goal of providing theoretical references for enhancing tea flavour.

## 2 Materials and methods

### 2.1 Experimental treatment and microorganism surface and metabolite sampling of tea leaves

The shed tea plants used in the experiment, *Camellia sinensis* cv. Huangshanzhong, were obtained from a two-year-old tea plantation in Xiazhuang Town, Ju County, Rizhao City, Shandong Province, China. Two acres of the tea plantation, where growth conditions and cultivation environment were consistent, were randomly selected for different material experimental treatments. All experimental tea plants were pruned before the foliar application.

The seaweed fertiliser used in this study was produced from waste seaweed by biological methods by Shandong Hengtai Marine Biotechnology Co. The composition of the seaweed fertiliser included 100.8 g/L of amino acid, 20.5 g/L of trace element (Cu, Fe, Zn, B, Mn), 1.5 g/L of water insoluble substance, and pH range of 3.0–9.0. Gibberellin was purchased from Jiangxi Xinruifeng Biochemical Co. Tea plants treated with gibberellin (CH) were used as the control group, while tea plants treated with seaweed fertiliser (CF) were placed in the experimental group. Seaweed fertiliser and gibberellin as foliar fertiliser were applied at rates of 100 mL and 100 g per 666 square metres of tea plants, respectively, diluted in the ratio of 3:1000, and applied at 7-day intervals for a total of 3 repetitions. The concentration of gibberellin (GA₃) referenced from (Li et al., 2021b) falls within the safe range. Fresh tea leaves with one bud and two leaves were collected. The fresh tea leaves were pre-treated and then stored in a − 80 °C refrigerator before undergoing Liquid chromatography-mass spectrometry (LC-MS) analysis, untargeted metabolomics, and 16S and ITS sequencing. LC-MS was performed in triplicate, while untargeted metabolomics and 16S and ITS sequencing were repeated five times.

### 2.2 Sensory evaluation

A panel of three certified tea evaluators conducted sensory evaluations according to the national standard GB/T23776–2018. The evaluation included five factors: appearance, tea infusion, aroma, flavour, and leaf characteristics. After picking, the tea leaves experienced killing (230–260 °C, 30 s), straightening (170–190 °C, 5 min), flattening (170–185 °C, 2 min), stir-frying (160–180 °C, 15 min), and aroma-raising 95 °C for 1 h until the dry tea aroma was obvious, with a greenish yellow colour, and then the production qs completed. The CF and CH tea samples were prepared with 100 g ∼ 200 g each and placed in the tea tray for evaluation. The evaluators held the tea tray by opposite corners with both hands and used the rotary sieve method to layer the tea samples sequentially. All the tea were assessed by visual inspection, touch, and other methods, repeating observation and comparison. We declared that ethical permission to conduct human sensory research was not a requirement in China. We will protect the rights and privacy of all participants during the research process by obtaining their consent and not forcing them to participate in the study. Do not release participant data without the participant's knowledge. Participants could withdraw from the study at any time. Consent to conduct the sensory evaluations described in this study was obtained from all members of the tasting panel. The panel members also confirmed that they consented to the use of their personal information and the publication of pertinent data.

For the tasting, 5.0 g of each CF and CH tea samples, were brew with 250 mL of boiling water, covered with a lid for 4 min, and then the tea infusion was filtered at an equal speed, leaving the leaves bottom in the cup. The sample were reviewed in order of tea infusion, aroma, flavour, and leaf bottom characteristics. The evaluation results were recorded using a percentage system, with the following weights for each factor: appearance 25 %, tea infusion 10 %, aroma 25 %, flavour 30 %, and leaf characteristics 10 %, respectively (Zhou et al., 2022).

### 2.3 Determination of flavour-related metabolites

The catechin (C), (−)-epicatechin (EC), epigallocatechin (EGC), gallocatechin (GC), (−)-catechin gallate, (−)-epicatechin gallate (ECG), (−)-epigallocatechin gallate (EGCG), gallocatechin gallate (GCG), L-theanine and caffeine (all >99 % pure, as determined through HPLC) were purchased from Aladdin (Shanghai, China). High performance liquid chromatography (HPLC) was performed to detect the quantitative data for 10 flavour-related metabolites including GC, EGC, C, EC, EGCG, GCG, ECG, CG, Caffeine, and L-theanine according to national standard methods (Fang et al., 2021).

### 2.4 Untargeted metabolomics measurements

Tea samples were placed in a 2 mL centrifuge tube with a 6 mm diameter grinding bead. Metabolite extraction was performed with 0.02 mg/mL of internal standard (L-2-chlorophenylalanine). The samples were ground in a frozen tissue grinder for 6 min (−10 °C, 50 Hz), followed by cryosonic extraction for 30 min (5 °C, 40 kHz). After standing at −20 °C for 30 min, the samples were centrifuged for 15 min (4 °C, 13,000 g), and the supernatant was pipetted into an injection bottle with an internal cannula for analysis. Equal volumes of sample metabolites were mixed to prepare a quality control (QC) sample to examine the reproducibility of the whole analytical process. LC-MS analysis was performed using a Thermo Fisher Ultra High Performance Liquid Chromatography Tandem Fourier Transform Mass Spectrometry UHPLC -Q Exactive system. An aliquot of sample (2 μL) was separated on a HSS T3 column (100 mm × 2.1 mm i.d., 1.8 μm) and then detected by mass spectrometry. The mass spectrometry signals were acquired in both positive and negative ion scanning modes, with a mass scanning range of 70–1050 *m*/*z*. The ion spray voltages were 3500 V for positive ions and 2800 V for negative ions.

### 2.5 Metabolomic dataset processing and analysis

LC-MS raw data were imported into metabolomics processing software Progenesis QI (Waters Corporation, Milford, USA) and matched using the metabolic public database HMDB (http://www.hmdb.ca/) to obtain metabolite information (Chang et al., 2023). The preprocessed matrix files were analysed for differences. The R package ropls (Version 1.6.2) was subjected to Principal Component Analysis (PCA) and Orthogonal Least Squares Discriminant Analysis analysis (OPLS-DA). In addition, student's *t*-test and multiplicative analysis of variance were performed. The selection of differential metabolites was determined based on the Variable Importance for the Projection (VIP) obtained from the OPLS-DA model and the *p*-value of student's t-test. Metabolites with VIP > 1 and *P* < 0.05 were considered differential. Differential metabolites were annotated by metabolic pathway annotation using the KEGG database (https: //www.kegg.jp/kegg/pathway.html) to obtain the pathways in which the differential metabolites were involved. Pathway enrichment analysis was performed using the Python package scipy.stats, and Fisher's exact test was applied to determine the most relevant biological pathways for the experimental treatments.

### 2.6 Microbial DNA extraction and sequencing

Tea leaves surface microbiota DNA was extracted by the E.Z.N.A.® soil DNA kit (Omega Bio-tek, Norcross, GA, U.S.). DNA quality was checked via 1 % agarose gel electrophoresis, and DNA concentration and purity were determined using a NanoDrop2000 (Thermo Scientific, U.S.A.). The 16S rRNA gene was nested, and the v5-v7 variable region was amplified in a second round using upstream primer 799F (5’-AACMGGATTAGATACCCKG-3′) and downstream primer 1193R (5’-ACGTCATCCCCACCTTCC-3′). A 2 % agarose gel was applied to recover PCR products, which were then detected and quantified by Qubit 4.0 (Thermo Fisher Scientific, USA). Libraries were constructed using the NEXTFLEX Rapid DNA-Seq Kit, and sequencing was conducted on the Illumina PE300 platforms (Shanghai Meiji Bio-pharmaceutical Technology Co.).

The double-end sequenced sequences were quality controlled using fastp (version 0.19.6) and were spliced using FLASH (version 1.2.11). Sequences were clustered using UPARSE (version 7.1) to generate operational taxonomic unit (OTU) after quality control and splicing. OTU species taxonomic annotation was performed using the RDP classifier (version 2.11) and compared to Silva 16S rRNA gene database (version 138). The 16S functional prediction analysis was conducted using PICRUSt2 (version 2.2.0).

### 2.7 Microbiological dataset processing

Alpha diversity was calculated by mothur. The similarity of microbial community structure between samples was tested using PCoA analysis based on the bray-curtis distance algorithm. The PERMANOVA non-parametric test was applied to determine whether the differences in microbial community structures between sample groups were significant. Bacterial taxa with significant differences in abundance from phylum to genus level between groups were identified using Linear discriminant analysis Effect Size (LefSe) analysis (LDA > 2, *P* < 0.05). KEGG metabolic pathway analysis was conducted to predict functional information of microbial communities between different groups, revealing potential functional characteristics through functional composition and abundance. Correlations between signature microbial communities and metabolites were analysed using Redundancy analysis/Canonical correspondence analysis (RDA/CCA) and correlation.

### 2.8 Statistical analysis

Analyses were performed in triplicate and data are expressed as mean ± SD. Data were analysed using GraphPad Prism 8.0.2. One-way ANOVA was employed to test for differences between groups, with *P* < 0.05 as the level of significance. Visualisation was performed using the packages on R 4.4.1.

## 3 Results and discussion

### 3.1 Sensory evaluation of tea leaves under different treatments

**Fig. 1:**
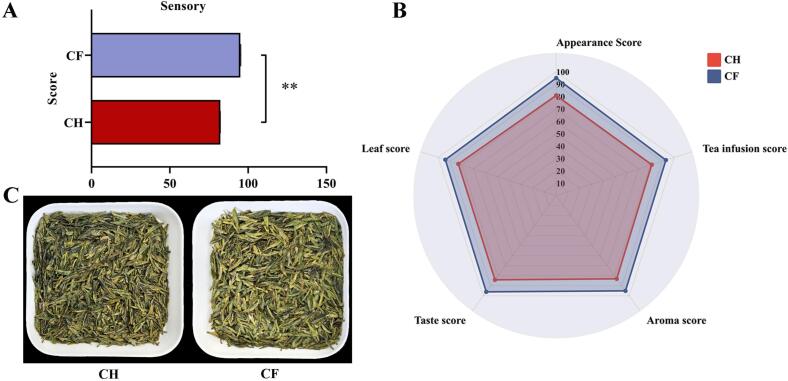
Sensory quality analysis of tea leaves of different treatments (A) Overall Score (B) Sensory evaluation (C) Dry tea appearance.

To investigate the effect of different treatments on tea quality, sensory evaluation of CH and CF samples was carried out (Fig. 1, Table S1). The CF scored higher than the CH in all five aspects of appearance, tea infusion, aroma, taste, and leaf (Fig. 1B). The overall quality score of CF was 95 (Fig. 1A). The taste of CF tea was umami, mellow and fragrant, and aroma had sweet, pure flavour characteristics. Whereas CH expressed slightly mellow and thick, slightly astringent with pure. Aroma and flavour are the dominant factors affecting the tea leaves quality. Astringency, in particular, influences the overall flavour of tea leaves (Dong et al., 2024). The CF tea achieves better flavour quality with enhanced aroma, reduced astringency, sweet, and mellow taste. The overall colour of tea leaves in CH exhibited darker effect than CF (Fig. 1C), which might be due to the differences in tea metabolites caused by different treatments. Flavonoids/flavonol glycosides are colour contributors to black tea infusion and drove colour transformation (Chen et al., 2024). Variations in yellow tea with different colouration are attributed to changes in gallated catechins and flavonoid glycosides (Wei et al., 2023). Therefore, the CH treatments can effectively influence the chemical structure of flavonoids/flavonol glycosides in the tea.

### 3.2 Characterisation of non-volatile metabolites in tea leaves

**Fig. 2:**
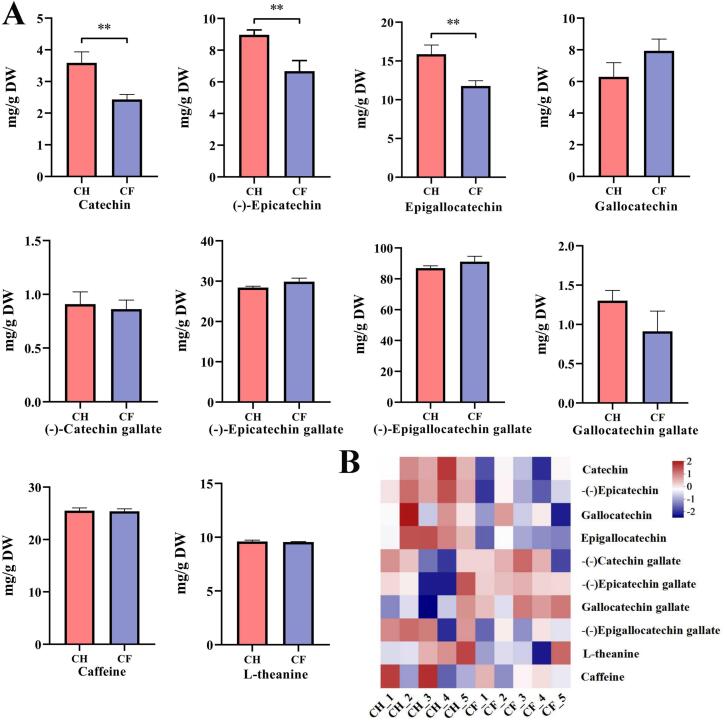
Quantitative and characterisation analyses of metabolomic data of tea leaves from CF and CH groups (A) Variations in content of 10 key flavour metabolites in quantitative data (B) Differences in content of 10 key flavour metabolites in untargeted metabolic data.

The flavour of tea depends on the amount and type of non-volatile compounds in the dry tea leaves (Shan et al., 2024). To identify the changes within the metabolites of shed tea under two treatments, the relative content of 10 key flavour metabolites were performed (Fig. 2A). All the 10 metabolites were detected in CF and CH tea samples. The metabolome data exhibited the same trend as the LC-MS data (Fig. 2B). Most flavour related substances appeared lower in CF than CH. Only Catechin, −(−)Epicatechin, and Epigallocatechin behaved notably with respect to CF and CH, and the levels in CF were all appreciably lower than the CH (*P* < 0.01). The flavour metabolites affect the tea leaves quality level. Among them, galloylated catechins accounted for three quarters of all catechins, including EC and EGC, which intensified the bitter and astringent flavour (Zhang et al., 2020). Flavour is the essential factor in assessing the quality of tea leaves. Sweet and fresh flavours are generally welcomed by consumers, whereas bitter and astringent flavours are often unacceptable (Ye et al., 2022). Reduced catechins content in CF could alter flavour and impact quality. The remarkable changes in catechin compounds caused by different treatments exert critical implications for the flavour of tea.

Untargeted metabolomics profiling of tea samples subjected to two distinct treatments identified 1409 metabolites, of which 336 were classified as differentially accumulated metabolites (DAMs) following pretreatment (Table S2). Metabolites levels were markedly separated between CF and CH groups. To further explore the differences between tea samples between CF and CH groups, the metabolic data were analysed by OPLS-DA. OPLS-DA demonstrated clear separation between the CF and CH sample groups. The compount1 of the OPLS-DA model was 36.8 % (Fig. S1A). Metabolites with abundance in the top 20 also varied notably between groups (Fig. S1B). Epigallocatechin, 5-p-Coumaroylquinic acid, Catechin and Epicatechin were primarily down-regulated in CF.

**Fig. 3:**
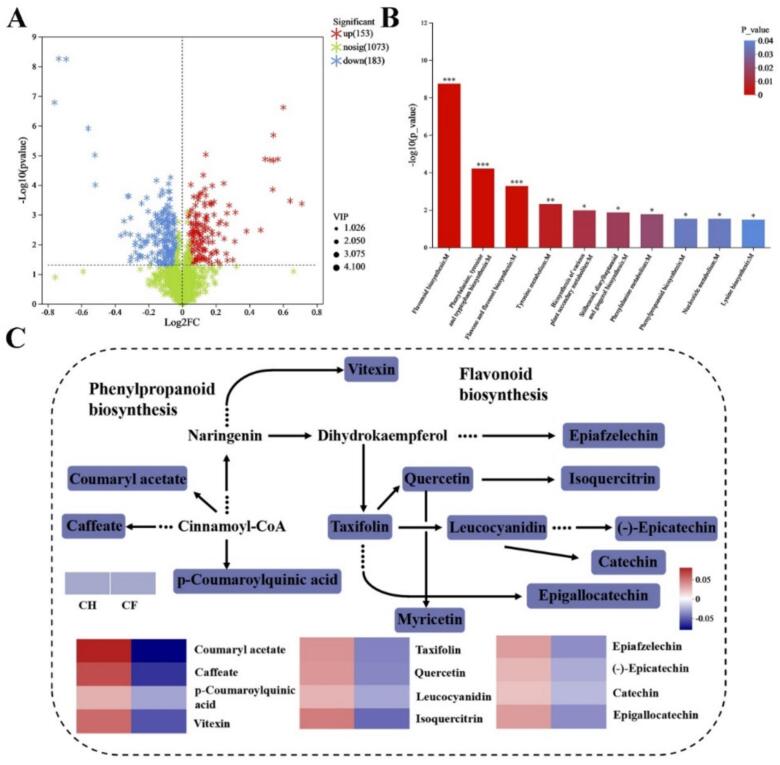
Metabolic pathways of differential metabolites (A) Volcano map of differential metabolites (B) The top10 significant KEGG-enriched pathway in DAMs (C) Phenylpropane and flavonoid biosynthetic pathways. Red indicated up-regulation, blue indicated down-regulation, and colour blocks in the pathway indicated differential metabolites. (For interpretation of the references to colour in this figure legend, the reader is referred to the web version of this article.)

In comparison with CH, 153 metabolites were notably up-regulated and 183 metabolites were prominently down-regulated in CF (Fig. 3A). KEGG pathway enrichment mapping by DAMs identified conspicuously enriched pathways including flavonoid biosynthesis, phenylalanine metabolism with phenylalanine, tyrosine, and plant secondary metabolites (Fig. 3B). The final metabolites of these pathways were chiefly catechin compounds (Singh et al., 2009). Metabolic pathways were mapped from DAMs versus enriched pathways. In particular, 13 DAMs were revealed in the metabolite enrichment of flavonoid biosynthesis and phenylpropanoid biosynthesis pathways, and all DAMs appeared down-regulated. Among the differing flavour metabolites were three monomeric catechins, (−)-Epicatechin, Epigallocatechin and Catechin, which emerged down-regulated in CF (Fig. 3C).

### 3.3 Composition and signature of bacterial and fungal communities in CF and CH

**Fig. 4:**
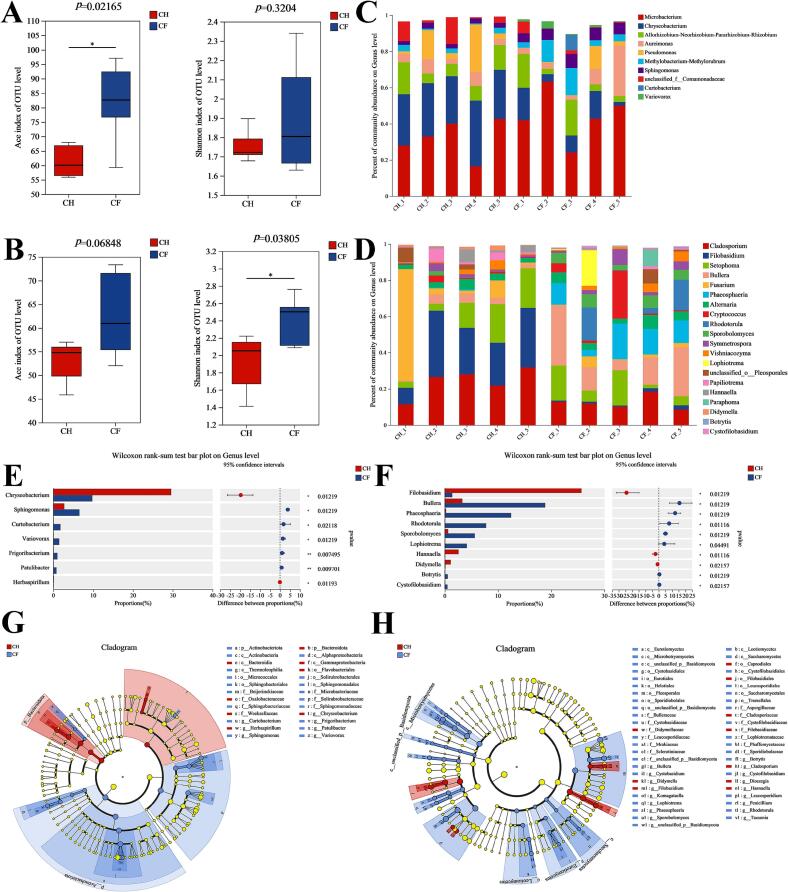
Multi-species variations and signature microorganism selection in CF and CH tea samples (A) Bacterial Ace and shannon index (B) Fungal Ace and shannon index (C) Species composition at the bacterial genus level (D) Species composition at the fungal genus level.

The 16S and ITS sequencing were taken to screen the CF and CH tea samples and the optimized validation sequences were obtained as 609,980 and 988,811, respectively. With 97 % similarity clustering, 672 and 566 OTUs were obtained, respectively (Table S3). The bacterial OTU population was 56–91 and 52–63 in CF and CH samples, respectively, while the fungal OTU was 51–73 and 45–57 in CF and CH samples, respectively. Common OUTs were observed in 77 and 60 in bacteria and fungi, respectively (Fig. S2 AB). With increasing amounts of extracted data, the sparse curve basically flattened out at the end, indicating that the amount of sequencing data was reasonable and sufficient (Fig. S2 CD). The Ace and Shannon indices in α-diversity illustrated that bacterial diversity was greater in CF than in CH and varied considerably in the Ace index. Similarly fungal diversity was greater in CF than CH and the Shannon index was dramatically different (Fig. 4 AB). Thus, both treatments altered the community structure and CF could increase the microbial community diversity.

Statistical community composition analysis of bacterial OTUs demonstrated a significant disparity in the relative abundance of Alphaproteobacteria at the taxonomic class level, constituting 34 % of total bacterial communities in the CF group versus 21 % in the CH group (Fig. S3A). Alphaproteobacteria and Thermoleophilia were substantially more abundant in CF than CH (Fig. S3C). Bacteroidia occupied 26–36 % in CH compared to 2–18 % in CF, with abundance levels notably lower than in CH. At the genus level, *Chryseobacterium* abundance was considerably higher in CH than CF, with a 3-fold difference, and might exist as a dominant bacterium (Fig. 4 CE). *Chryseobacterium* might potentially threaten the survival of other microorganisms, leading to a decrease in CH diversity. *Chryseobacterium* in Wuyi rock tea affected the production of epicatechin gallate, similar to Epigallocatechin in CH, and probably promoted the accumulation of catechin substances (Wu et al., 2024). Whereas *Sphingomonas*, *Curtobacterium* and *Variovorax* abundance were dominant in CF, which might contribute to the potential regulation of CF quality (Fig. 4E). *Sphingomonas* and *Variovorax* are the dominant genus in black tea processing (Jia et al., 2022). Bacterial abundance is intimately associated with epigallocatechin, epicatechin gallate, and catechin gallate, thus affecting black tea quality. In addition, both possessed antagonistic roles against pathogens, which likely favoured protection against external disturbances to tea (Li et al., 2022). The single bacterial genus *Variovorax* could furthermore improve colonisation in complex microbial communities and possibly be involved in colonising microbial communities that favoured tea quality metabolites (Finkel et al., 2020).

At class level of fungi Microbotryomycctes constituted only 0.6 % in CH, while in CF was 13 %, which resulted obvious difference (Fig. S3B). The abundance of *Filobasidium* at the genus level was considerably higher in CH than CF (26 % vs. 1 %). *Filobasidium* emerged as the dominant fungus in wild tea plants and was notably enriched in flavonoid and flavonol biosynthesis pathways (Zhang et al., 2022), similar phenomenon was also detected in CH. *Bullera* as a beneficial genus abundance was about six-fold higher in CF than CH (Fig. 4DF), which possessed antifungal activity as well as growth promoting activity (Khwantongyim et al., 2021). Besides, *Phaeosphaeria* and *Rhodotorula* might act as key colonies.

(E) Multi-species difference test at bacterial genus level (F) Multi-species difference test at fungal genus level (G) Signature bacteria LEfSe analysis (H) Signature fungi LEfSe analysis.

To further explore the differences between CF and CH tea microbiota, principal coordinate analysis was employed to reveal significant differences in both bacterial and fungal communities (Fig. S4). The signature bacteria and fungi in CF and CH were identified using LEfSe analysis (Fig. 4GH).

*Sphingomonas*, *Variovorax*, *Curtobacterium*, and *Frigoribacterium* were signature bacterial communities at the genus level of CF. *Bullera*, *Phaeosphaeria*, *Rhodotorula*, *Sporobolomyces* and *Lophiotrema* were genus level signature fungal communities. *Chryseobacterium*, *Filobasidium*, *Herbaspirillum* were signature microbial communities at the genus level in CH. Bacteroidota, Weeksellaceae were the phylum level and family level signature bacterial communities in CH, respectively. Filobasidiales and Capnodiales were order level signature fungal communities. The presence of signature microorganisms in form of two treatments could contribute to the identification and improvement of flavour variations.

### 3.4 Comprehensive analysis of microorganisms and metabolites

**Fig. 5:**
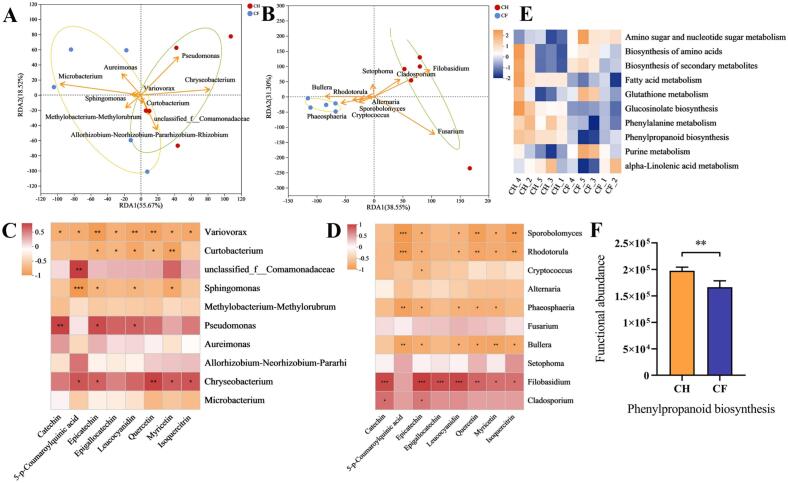
Correlation and functional analysis of microorganisms with metabolites (A) The RDA analysis of bacteria with metabolites (B) The RDA analysis of fungi with metabolites (C) Correlation analysis of bacteria with metabolites (D) Correlation analysis of fungi with metabolites (E) Functional analysis of KEGG pathway in bacteria (F) Differences in functional abundance of microorganisms in Phenylpropanoid biosynthesis.

RDA/CCA and correlation analyses were performed for the effects of different treatments on metabolite distribution (Fig. 5A-D). *Chryscobacterium*, *Pseudomonas*, and *Curtobacterium* exerted the active role in the metabolite's distribution in CH group, with *Chryscobacterium* acting the most vital role. *Microbacterium* functioned negatively with *Sphingomonas*. Correlation analysis revealed that *Pseudomonas* and *Chryscobacterium* were positively associated with metabolites content in the phenylpropanoid pathway. *Pseudomonas* and *Variovorax* performed notable positive and negative regulatory roles in characteristic secondary metabolites Catechin and Epicatechin, respectively. Catechin emerged as most powerfully positively correlated metabolite with *Pseudomonas*.

Besides, fungi *Bullera*, *Phaeosphaeria*, and *Rhodotorula* exerted key roles in CF group, with *Bullera* having the dominant role. *Filobasidium* dominated in CH group. Fungi *Filobasidium* and *Bullera*, *Rhodotorula*, and *Phaeosphaeria* were positively and negatively correlated with metabolites, respectively. *Filobasidium* displayed the highest correlation with Epicatechin, followed by Leucocyanidin, Catechin and Epigallocatechin. *Sphingomonas*, *Variovorax* and *Bullera*, *Rhodotorula*, and *Phaeosphaeria* might relate to variations in flavour metabolites for improving quality. The bacteria can be further inoculated to improve the quality. *Sphingomonas* within Fu Brick tea  played a pivotal role in metabolite evolution during fermentation (Wu et al., 2023), and *Sphingomonas* in CF might improve the quality by decreasing the Catechin level.

Analysis of KEGG metabolic pathways in the microbial community indicated that Phenylalanine metabolism, Phenylpropanoid biosynthesis, Glucosinolate biosynthesis, and Fatty acid metabolism functions were enriched in CH group (Fig. 5E). Microbial functions also varied considerably in Phenylpropanoid biosynthesis (Fig. 5F). The up-regulation of these pathways might attribute to the enrichment of microorganisms in CH, favoring the accumulation of flavonoid metabolites. The lower abundance of bacterial communities in CH probably related to the production of down-regulated synthases and up-regulated substance-associated degradative enzymes upon accumulation of flavonoid compounds. The function in CF was concentrated in amino sugar nucleotide sugar metabolism and purine metabolism. The biosynthetic pathways of amino acids and secondary metabolites were enriched to higher extent than in CH, which might somewhat promote the increase of amino acid substances and secondary metabolites.

In both treatments, the structure and composition of the microbial community were affected by the variations in inclusions of the treatments, which might lead to differences in the dominant bacteria and fungi. Differences in microbial composition, including genera such as *Chryscobacterium*, *Filobasidium*, *Sphingomonas*, *Variovorax*, and *Bullera*, may correlate with the metabolism of those flavouring substances. Due to those microbial variations, microbes with different functions performed diverse roles in enrichment, resulting in variable tea metabolites (Yan et al., 2024). The levels of catechin flavour-related metabolites were lower in CF, and the untargeted metabolome disclosed that the metabolites were distinctly dissimilar in CF and CH. The KEGG pathway analysis indicated multiple enrichment in metabolites associated with Flavonoid biosynthesis and Phenylpropanoid biosynthesis in CH. The improvement of tea flavour might depend on the enrichment of exogenous substances or flavour-related microbes, which together regulate changes in flavour metabolites. Reduced levels of catechin metabolites contributed to the improvement of flavour quality and also benefited human health (Wang et al., 2024).

## 4 Conclusions

The metabolites and microbial communities of tea leaves under CF and CH were studied. Sensory evaluation indicated that CF had a better taste. Three flavour metabolites, Cathchin, −(−)Epicatechin and Epigallocatechin were lower in CF. Untargeted metabolomics indicated DAMs concentrated in Flavonoid biosynthesis, Phenylpropanoid biosynthesis pathways. *Chryscobacterium* played a crucial role in the distribution of CH metabolites. *Variovorax* served as the notable negative regulator of the characteristic secondary metabolites Catechin and Epicatechin. The Fungi *Bullera* and *Filobasidium* were key contributors in CF and CH, respectively. Besides, microorganisms in CF and CH exhibited diverse functional differences. Microbial communities contributed towards the tea quality improvement and involved in metabolic activities through various metabolites that formed the characteristic tea flavour. This comprehensive analysis provides valuable insights for improving greenhouse tea quality and deepens the understanding of the mechanisms influencing flavour in greenhouse tea cultivation and production. However, further research is needed to elucidate the potential mechanisms by which microbial interventions can alter the sensory attributes and quality of greenhouse tea.

## CRediT authorship contribution statement

**Haozhen Li:** Writing – original draft, Methodology, Investigation, Funding acquisition, Data curation. **Shuyao Wang:** Writing – review & editing. **Xiaohua Zhang:** Writing – review & editing. **Kangkang Song:** Writing – review & editing. **Long Yang:** Writing – review & editing, Writing – original draft, Supervision, Project administration, Investigation, Funding acquisition, Data curation, Conceptualization.

## Funding

This work was supported by the Foundation of Innovation Team Project for Modern Agricultural Industrious Technology System of Shandong Province (SDAIT-25-01) and Special Funds for Local Scientific and Technological Development Guided by the Central Government (YDZX2022123). And we thank Supercomputing Center in Shandong Agricultural University for technical support.

## Declaration of competing interest

The authors declare that they have no known competing financial interests or personal relationships that could have appeared to influence the work reported in this paper.

## Data Availability

Data will be made available on request.

## Appendix A Supplementary data

Supplementary material 1 Image 1

Supplementary material 2 Image 2

Supplementary material 3 Image 3

Supplementary material 4 Image 4
