# Supplementary material for: Revealing novel insights into the improvement of greenhouse tea quality through exogenous substance interventions using targeted and untargeted metabolomics and microbial community analyses
Source: Food Chem X. 2025 Mar 24;27:102410. doi: 10.1016/j.fochx.2025.102410 (PMC11985128; doi:10.1016/j.fochx.2025.102410)
Supplement: Supplementary file 1 — Supplementary material 1 [file mmc1.docx]

Revealing novel insights into the improvement of greenhouse tea quality through exogenous substance interventions using targeted and untargeted metabolomics and microbial community analyses

Haozhen Li^1^, Shuyao Wang^1,2^, Xiaohua Zhang^1^, Kangkang Song^1^ and Long Yang^1^*

^1^College of Plant Protection, Agricultural Big-Data Research Center and Key Laboratory of Agricultural Film Application of Ministry of Agriculture and Rural Affairs, Shandong Agricultural University, Tai’an 271018, China

^2^Bioresource Engineering, McGill University, Sainte-Anne-de-Bellevue, QC, H9X 3V9, Canada

* Correspondence: lyang@sdau.edu.cn

The following are additional figures:


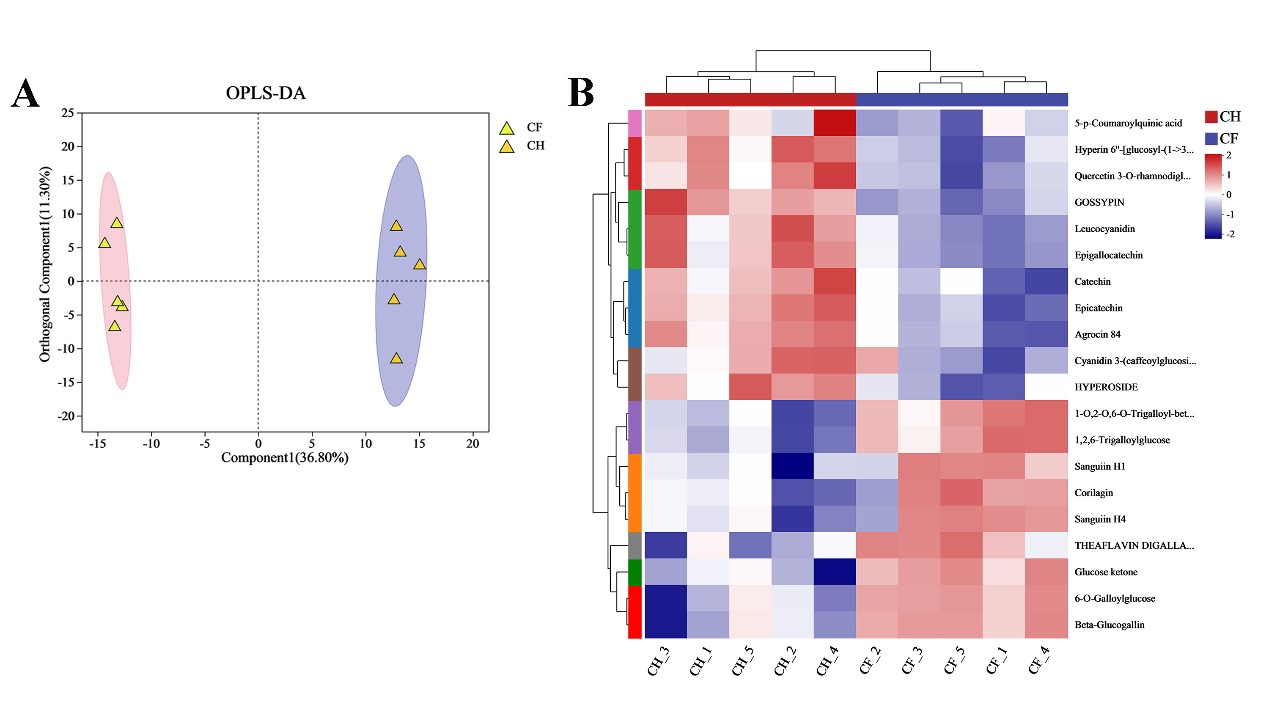


Fig. S1 Metabolomic characterisation of tea leaves from CF and CH (A) OPLS-DA modelling of tea samples from different treatments (B) Heatmap of clustering with abundance in top 20 metabolites


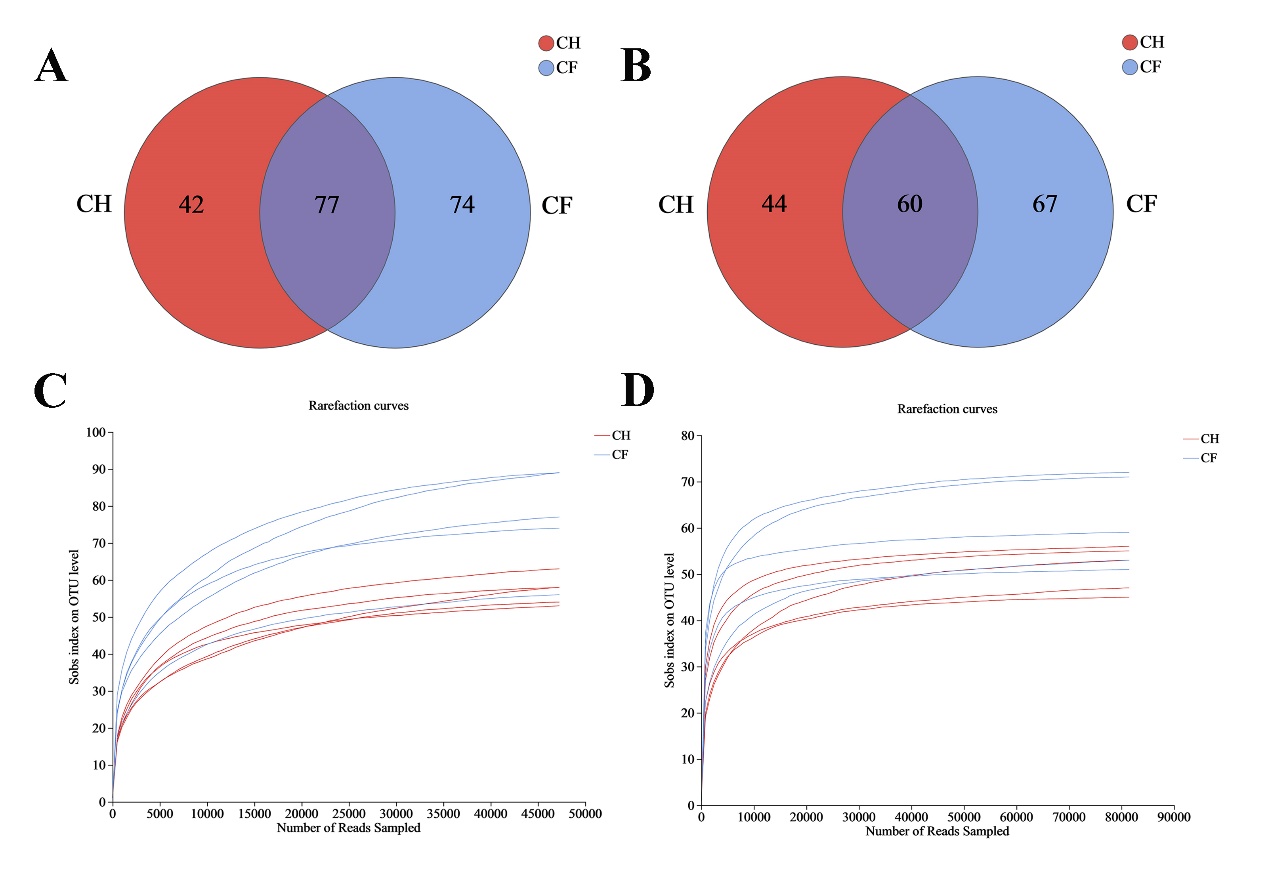


Fig. S2 Sequencing data characteristics of microorganisms (A) Number of bacterial OTUs (B) Number of fungal OTUs (C) Bacterial sparsity profile (D) Fungal sparsity profile


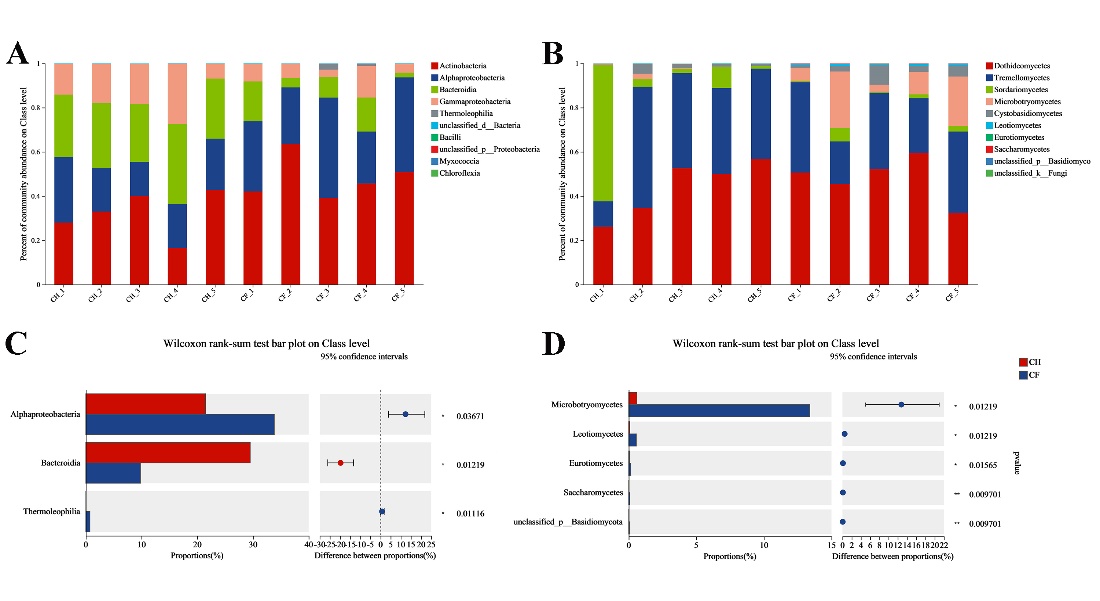


Fig. S3 Multi-species variations in CF and CH tea samples (A) Species composition at the bacterial class level (B) Species composition at the fungal class level (C) Multi-species difference test at bacterial class level (D) Multi-species difference test at class genus level


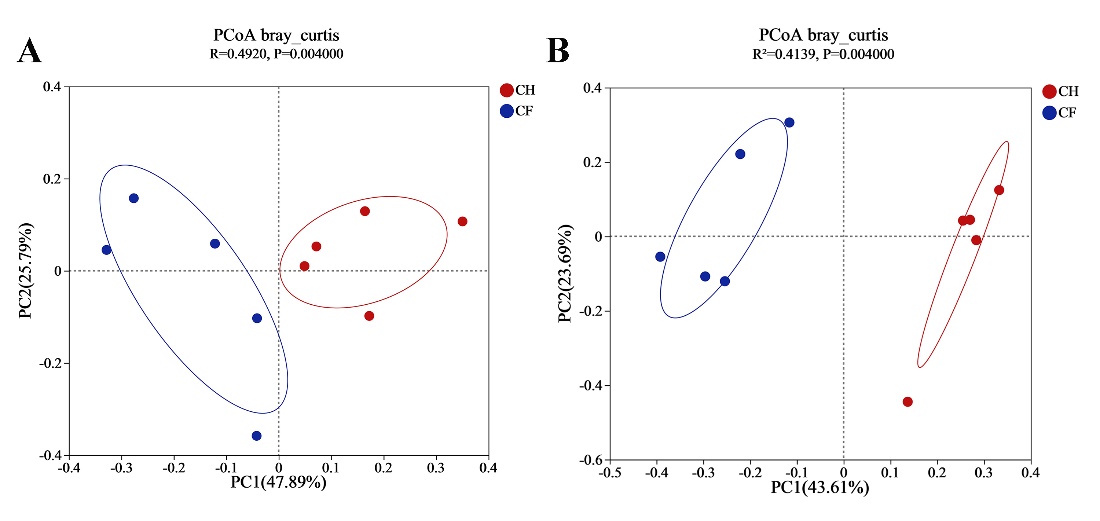


Fig. S4 Differences in microbial communities (A) principal coordinate analysis in bacterial (B) principal coordinate analysis in fungal
